# Tumor Delivery of Chemotherapy Combined with Inhibitors of Angiogenesis and Vascular Targeting Agents

**DOI:** 10.3389/fonc.2013.00259

**Published:** 2013-10-01

**Authors:** Marta Cesca, Francesca Bizzaro, Massimo Zucchetti, Raffaella Giavazzi

**Affiliations:** ^1^Laboratory of Biology and Treatment of Metastases, Department of Oncology, IRCCS-Istituto di Ricerche Farmacologiche “Mario Negri”, Milan, Italy; ^2^Laboratory of Cancer Pharmacology, Department of Oncology, IRCCS-Istituto di Ricerche Farmacologiche “Mario Negri”, Milan, Italy

**Keywords:** combination therapies, angiogenesis inhibitors, tyrosine kinase receptor inhibitors, vascular disrupting agents, drug delivery, paclitaxel, tumor microenvironment

## Abstract

Numerous angiogenesis-vascular targeting agents have been admitted to the ranks of cancer therapeutics; most are used in polytherapy regimens. This review looks at recent progress and our own preclinical experience in combining angiogenesis inhibitors, mainly acting on VEGF/VEGFR pathways, and vascular targeting agents with conventional chemotherapy, discussing the factors that determine the outcome of these treatments. Molecular and morphological modifications of the tumor microenvironment associated with drug distribution and activity are reviewed. Modalities to improve drug delivery and strategies for optimizing combination therapy are examined.

## Introduction

Recognition of the multi-compartment nature of the tumor microenvironment has challenged the conventions of anticancer therapy, giving rise to a radically different approach toward the discovery of new treatments. Historically aimed at killing tumor cells (cytotoxic agents), the search now seeks to identify novel “biologicals,” that selectively target not only the cancer cell, but also the tumor stroma.

Angiogenesis – the development of new vasculature – is required for tumor growth, invasion, and metastatic dissemination, hence the rationale for anti-angiogenic therapy (1). Numerous angiogenesis-targeting agents have been admitted to the ranks of cancer therapeutics (2). Drugs targeting the tumor vasculature have been developed and have shown efficacy in preclinical models and in some clinical trials (1–3).

The most validated anti-angiogenic strategy to prevent tumor vessel formation targets the vascular endothelial growth factor (VEGF) axis. VEGF can be blocked directly, with the antibody bevacizumab (Avastin^®^), or, among others, with the VEGF-trap (Aflibercept^®^), an engineered soluble VEGF receptor able to bind VEGF as well as platelet growth factor (PLGF) (4), or indirectly by inhibiting receptor activity with antibodies or small-molecule tyrosine kinase receptor inhibitors (RTKIs). Sunitinib (Sutent^®^), sorafenib (Nexavar^®^), and pazopanib (Votrient^®^) have been approved for different tumor types (5). An alternative strategy is to selectively destroy the existing vasculature with vascular disrupting agents (VDA) (6–8). VDA cause a pronounced and rapid shutdown of blood flow to solid tumors, leading to tumor necrosis and death. Small molecules, flavonoids (DMXAA), and tubulin-binding agents (Ca4P, ZD6126, Ave8062, Oxi4503), have entered into Phase II–III studies.

Inhibition of a single target or pathway is of limited benefit for cancer patients (9). The primary clinical use of bevacizumab is combined with chemotherapy, as only the combination significantly prolonged overall survival in patients with metastatic colorectal cancer (CRC) (10) and recurrent/advanced non-small cell lung cancer (NSCLC) (11), or extended progression-free survival in patients with advanced ovarian cancer (12–14) and renal cell cancer (15). Unlike bevacizumab, the multitargeted profile of RTKIs makes them active as single agents, and any clear advantage in combination with chemotherapy has yet to be demonstrated (16).

The mechanism by which anti-angiogenic agents increase the efficacy of chemotherapy is not yet clear. An angiogenesis inhibitor combined with chemotherapy affects multiple compartments, depriving the tumors of nutrients and oxygen (i.e., anti-vascular and anti-angiogenic effect), and killing highly proliferative tumor cells (i.e., cytotoxic effect) (17). In terms of drug delivery this sounds paradoxical since the anti-angiogenic therapy, by modifying the tumor vasculature, potentially impairs the delivery of cytotoxic drugs (18).

The tumor microenvironment has an abnormal vasculature, structurally and functionally (increased vessel permeability, dilatation and tortuosity, reduced pericyte coverage, and abnormal basement membranes), mainly because of an imbalance between pro- and anti-angiogenic factors (19, 20). As a consequence, tumor blood flow is impaired and this, together with compression of the blood vessel by the growing cancer, results in high interstitial fluid pressure (IFP), hypoxic regions within the tumor, and ultimately reduced drug delivery (21, 22).

Anti-VEGF, and more in general anti-angiogenesis agents, modify the tumor microenvironment; abnormal microvessels are destroyed and the remaining vessels are remodeled (2). These changes, that led Jain and coworkers to formulate the hypothesis of “vascular normalization,” should lead to a transient increase in vascular patency, a drop in IFP and alleviation of hypoxia, providing a “window of opportunity” for the delivery of drugs, with better therapeutic outcome. For reviews on tumor microenvironment normalization see Jain (23, 24). The normalization hypothesis offers a solution to the paradox that some angiogenesis inhibitors are efficacious when combined with chemotherapy.

Whether these morphological changes are accompanied by functional modifications, such as improved drug delivery, is still debated (25–27). Current attempts at combination treatments are often empirical, though rational protocols are needed that take account of drug pharmacokinetics, and their metabolic interactions and mechanism of action, as well as the biological characteristics of the tumor microenvironment (28). Careful dosing, scheduling, and sequencing of treatments, to avoid possible negative interactions and side effects, become essential to optimize the efficacy of combinations (7, 29). Optimization requires reliable, robust end points to monitor the activity of the combination.

This review focuses on recent progress, and our own preclinical experience in combining angiogenesis inhibitors/vascular targeting agents with conventional chemotherapy. Molecular, morphological and functional modifications of the tumor microenvironment related to drug distribution and activity are reviewed and, we examine some modalities to improve drug delivery and strategies for the optimization of combination therapy.

## Combination with Bevacizumab

Bevacizumab is a humanized monoclonal antibody that targets VEGF (30). It has been approved, in combination with chemotherapy, for a number of malignancies, including colon, lung, and ovarian (in Europe) cancer (10–12), and for kidney cancer combined with interferon-gamma.

According to the hypothesis of vessel “normalization,” Jain and coworkers showed in a number of tumor models transplanted in the cranial window or in the dorsal skinfold chamber of mice, that vessels begun to function better when treated with a neutralizing antibody anti-VEGF, possibly enhancing delivery of chemotherapy (31). Next, the duration of the “normalization window” was associated with alleviation of hypoxia, which plays a role in drug resistance and tumor progression. Radiotherapy was also more effective when administered during the normalization window (32). However, while preclinical studies have reported morphological and functional changes in the tumor vasculature after blocking VEGF, studies on drug delivery after anti-angiogenesis treatment are scanty. A tendency to a higher CPT11 concentration, that paralleled increased tumor perfusion, was observed in a colon carcinoma growing in nude mice after VEGF-blocking therapy. This suggested an increase in transport capability by vessels surviving anti-angiogenic treatment that compensated the reduction in the number of patent blood vessels (33).

The main concern about the “normalization window” is the limited time in which it occurs. As an example, orthotopic neuroblastoma xenografts treated with bevacizumab were evaluated at serial time points for treatment-associated changes in intratumoral vascular physiology, penetration of chemotherapy, and efficacy of combination therapy. After bevacizumab, there was a progressive decrease in tumor microvessel density, with a rapid, sustained fall in tumor vessel permeability and tumor IFP, while tumor perfusion (mirrored by drug delivery) improved. Unfortunately these changes were short-lasting; the improvement in drug delivery was observed only for a few days after bevacizumab, but not when both drugs were given concomitantly. Although the combination was always superior to single-agent treatment, sequential treatment within the “normalization window” gave no significant advantage over concomitant treatment (34).

Our laboratory found that bevacizumab in combination with chemotherapy delayed tumor progression in mice bearing ovarian carcinoma xenografts, significantly prolonging survival (35). We observed a clear effect of bevacizumab on vessel morphology toward a “normalization” phenotype (e.g., decrease in vessel number, increase in pericyte coverage), but this was not related to increased drug uptake into tumor. While these findings do not explain the better outcome with the combination, we hypothesize that after bevacizumab, distribution of the cytotoxic drug might be better in more vital and actively proliferating areas of the tumor. Studies are in progress using Imaging Mass Spectrometry to clarify the spatial distribution of drugs into the tumor tissue after angiogenesis inhibitors (36).

Similar considerations can be extended to the combination of bevacizumab with large molecules, such as antibodies. For example reduced uptake of trastuzumab (a monoclonal antibody directed against HER-2/neu) after bevacizumab was observed in HER-2 expressing breast cancer xenografts. This was presumably due to tumor blood flow and vascular permeability reduction which contribute to the changes of trastuzumab pharmacokinetics (37). However the combination of bevacizumab with trastuzumab has given promising results in breast cancer patients (38).

Few clinical studies report the effects of anti-angiogenic therapy on drug uptake. One of the first pointers to the anti-vascular effect of bevacizumab in a clinical setting, came from a phase I study, in which patients with non-metastatic CRC were given a single infusion of bevacizumab, concomitantly with neo-adjuvant chemotherapy with 5-fluorouracil. Twelve days after its infusion, bevacizumab reduced tumor blood perfusion and vascular volume, accompanied by decreases in microvessel density, lower IFP, and increased pericyte coverage, confirming the drug’s anti-vascular and normalizing effects in human tumors (39). Vessel permeability, assessed as computed tomography (CT) contrast agent extravasation, and fluorodeoxyglucose (FDG) uptake into the tumor during positron emission tomography (PET) scans, did not change, indirectly showing cytotoxic drug uptake was not affected. PET scan 6 weeks after the bevacizumab and chemotherapy showed reduced FDG uptake than in previous scans, in accordance with the “temporary” duration of the normalization window (39).

To elucidate the effects of angiogenesis inhibitors on drug delivery, a recent study used PET to investigate bevacizumab combined with (^11^C)docetaxel in NSCLC patients. Bevacizumab reduced perfusion and the net influx rate of docetaxel, shortly after its administration, and for several days afterward, showing no substantial improvement in drug delivery into tumors. Interestingly, bevacizumab prolonged systemic drug exposure, reducing plasma clearance, and causing more homogeneous intratumoral distribution, as a result of some normalization of tumor vasculature (40). These findings indicate that anti-VEGF therapy not only does not improve tumor drug delivery, but rather has an opposite effect. The authors also suggested that the anti-angiogenic drug might be given after the anticancer agent, as the immediate decrease in tumor perfusion should reduce the clearance of drugs from tumors.

Thus, there is currently a large body of evidence indicating that agents such as bevacizumab cause vascular normalization, but whether this phenomenon favors or not drug penetration into tumors remains unclear. Studies in humans highlight the importance of drug scheduling and call for further studies to optimize combination modalities (41).

## Combination with Receptor Tyrosine Kinase Inhibitors

A large number of small molecules that are multi-receptor RTKI, have progressed through clinical development (5). Unlike bevacizumab, the RTKIs have antitumor activity in monotherapy, and are rarely used in combination with chemotherapy in clinical practice, as no clear benefit has been reported (16). Clinical data on chemotherapy uptake after RTKIs are lacking.

The partial advantage we have observed, in preclinical models combining sunitinib (VEGFRs, PDGFRs, and c-Kit inhibitor) with chemotherapy (42) raises the question whether these molecules differ from bevacizumab in their anti-angiogenic actions and herein in their ability to facilitate drug distribution and activity.

Several preclinical studies have exploited RTKIs in combination with chemotherapy aimed to study drug interaction and modification of the tumor/stroma compartment which can ultimately affect drug distribution. RTKIs often give rise to increased hypoxia and decreased drug uptake [for review see Ref. (43)].

For example, the small-molecule axitinib (VEGFRs, PDGFR, and c-KIT inhibitor) affected tumor vasculature as the number of blood vessels decreased, and hypoxia increased (44, 45). In combination with cyclophosphamide, this led to decreased delivery of its metabolite (4-OH-CPA) into the tumor, with the consequence of limited antitumor activity, and no tumor regressions (44). Interestingly, the anti-vascular activity of axitinib, could be turned to a therapeutic advantage if cyclophosphamide was injected intra-tumor: axitinib slowed leakage of 4-OH-CPA, increasing its retention (46). Thus drug retention, as consequence of anti-angiogenic therapy, can be exploited for combination with drugs injected intra-tumor or for systemic delivery of pro-drugs directly activated in the tumor (46). (See also combination with VDA below).

Some years ago we studied the combination of SU6668 (a first-generation VEGFR-2, FGFR, and PDGFRβ inhibitor) with paclitaxel on ovarian cancer xenograft models (47, 48). The combination affected tumor burden and prolonged overall survival, depending on the tumor’s sensitivity to paclitaxel (less efficient on the resistant tumor), the treatment regimen (less active, though less toxic at a metronomic schedule) and the tumor burden at the beginning of treatment (less active on large tumors). Though SU6668 alone and in combination affected tumor vascular density (48), there was no improvement in paclitaxel uptake. The limited advantage given by SU6668 added to paclitaxel on the resistant tumor compared to the sensitive one indicated that angiogenesis inhibitors and cytotoxic agents act on the tumor and host compartments independently, with some combined effects on the same compartment (the host), as paclitaxel is a strong vascular targeting drug (49).

These initial findings prompted us to investigate morphological and functional changes of the tumor vasculature induced by the RTKI vandetanib (VEGFR2, EGFR, and RET inhibitor), and its effect on intratumoral delivery and the antitumor activity of paclitaxel (27). In line with previous observations, the combination of vandetanib plus paclitaxel had greater antitumor activity than the single agent treatments (50). However, changes in vascular morphology and function (normalization) induced by vandetanib were not associated with any increase in paclitaxel delivery into the tumor. In fact, our results showed that the antitumor activity of vandetanib combined with paclitaxel is at least partly dependent on the drug sequence. In mice pretreated with vandetanib, paclitaxel delivery decreased, reflecting a decrease in tumor perfusion, assessed as Hoechst 33342 levels, an indicator of vascular perfusion (27). The decrease in uptake of paclitaxel after vandetanib was particularly evident at an early time point (1 h after paclitaxel) as levels were similar later (24 h after paclitaxel). As plasma data excluded reduced drug availability, this might have been due to less efficient paclitaxel penetration in the more poorly perfused vandetanib-treated tumor, followed by longer retention for the same reason. Vandetanib impaired paclitaxel uptake already after 1 day of treatment, with maximum effect after 5 days. On stopping vandetanib, paclitaxel uptake by the tumor was restored, indicating that vandetanib’s effect on drug distribution in tumors is reversible as it is the “normalization” phenomenon (27).

We have observed reduced paclitaxel delivery into the tumor with no change in systemic pharmacokinetics, in combination experiments with different RTKIs (sunitinib and the dual inhibitors of VEGFR2 and FGFR2, brivanib and E-3810), despite the improved antitumor activity. E-3810 reduced vessel number, and induced tumor microenvironment modifications which ultimately lead to remodeling of the extracellular matrix (51).

At variance, inhibition of PDGFR signaling with an increase in taxol uptake into the tumor and greater therapeutic effect have been described (52). Imatinib, beside its original selectivity for Bcr-Abl tyrosine kinase, affects PDGFRβ, lowers IFP and microvessel density, and improves tumor oxygenation, consequently increasing the tumor concentration of small molecules such as docetaxel, or bigger ones such as liposomal doxorubicin (53).

One possible explanation for these controversial findings could be the different inhibitors of angiogenesis used in those study. Also the dose of the anti-angiogenic drug, might play an important role, as a too high dose can cause too rapid vessel pruning and not favor drug delivery and anticancer effects (24). The effect of different doses of RTKI on vessel morphology and functionality was not explored in the above study.

In the light of these considerations, we suggest that the combination of RTKI with chemotherapy is feasible. A better use of pharmacodynamics and pharmacokinetics, might help to maximize the effect and avoid negative interactions of RTKI combined with chemotherapy. Recently we have shown that the addition of certain chemotherapeutics to sunitinib is able to counteract the unwonted negative effect on tumor metastasization (42).

## Combinations with Other Angiogenesis Inhibitors

The first combination modalities based on anti-angiogenic compounds used TNP-470, an inhibitor of methionine aminopeptidase, an essential enzyme for endothelial cell proliferation. TNP-470 potentiated the antitumor activity of cytotoxic therapeutics, increasing their biodistribution into tumor tissue, an effect that was sufficient *per se* to account for the greater delay in tumor growth (54).

A number of molecular targets, alternatives to VEGF/VEGFR and related growth factors, implicated in vascular remodeling, are worth considering for the development of novel therapeutic modalities.

Semaphorin 3A (Sema3A) is expressed in endothelial cells, where it serves as an endogenous inhibitor of angiogenesis, and is lost during tumor progression. Its long-term re-expression at a later stage of carcinogenesis stably normalized the tumor vasculature in transgenic mouse tumor models and impaired tumor growth (55). In an accompanying study the authors showed there were larger amounts of doxorubicin in Sema3A-treated tumors, than controls, so Sema3A re-expression substantially extends the normalization window of tumor blood vessels and improves the delivery efficiency of chemotherapeutic drugs (56).

Selective killing of tumor neovasculature with an antibody directed against tumor vascular endothelial VE-cadherin, conjugated with an α-particle-emitting isotope generator, caused vascular remodeling, increased tumor delivery of chemotherapy, and reduced tumor growth. Interestingly, the effect was seen when chemotherapy was scheduled several days after the anti-vascular therapy. The authors pointed out that after depletion of the majority of vessels, the remaining ones appear more mature, so small-molecule drugs more homogeneously distribute and accumulate better, as reflected in the improvement of antitumor activity (57).

## Combination with Vascular Targeting Agents

Therapeutic vascular targeting agents comprise small molecules, mainly tubulin-binding agents, flavonoids, antagonists of junctional proteins intended to selectively target the tumor vasculature (VDA), and compounds that target proteins expressed selectively on tumor vasculature used to deliver bioactive molecules (6, 58, 59). VDA induce morphologic changes in endothelial cells, triggering a cascade of events that results in rapid reduction of blood flow, and vessel occlusion, with subsequent tumor cell death. The hallmark of VDA action is the induction of massive central necrosis of tumor tissues, leaving a rim of viable, actively proliferating cells at the periphery of the lesion. The ability of these proliferating cells to repopulate the tumor explains the limited activity of these agents as monotherapy, but also justifies their use in combination with cytotoxic drugs. IFP levels dropped rapidly after VDA (60) suggesting that if they are used appropriately in conjunction with other drugs the efficacy of treatment may be enhanced. The benefit from such combinations should be complementary, with the VDA acting primarily on the tumor vasculature, and the chemotherapy mainly affecting proliferating tumor cells.

A number of VDA have reached the clinical stage (61). Their effects on tumor vasculature have obvious implications in the design of combination treatments given their possible interference with distribution of the cytotoxic drug (62). The sequence of administration has to take into account that the vessel shutdown induced by the VDA given after the cytotoxic compound would trap it within the tumor, at the same time preventing the possible VDA-induced impairment of drug distribution in the tumor. Conversely, the opposite schedule, i.e., the VDA before the cytotoxic drug, might generate favorable conditions for its activity because the highly proliferating cells at the periphery of VDA-treated tumors are an ideal target for cytotoxic drugs (7).

We administered the VDA ZD6126 followed by paclitaxel 24–72 h later; this combination had greater antineoplastic activity than each single agent, leading to complete tumor remissions (63). That study showed a significant increase in proliferative activity at the tumor periphery after ZD6126, concomitant with the induction of massive necrosis. It is therefore conceivable that pretreatment with ZD6126 affects the inner part of the tumor, while chemotherapy targets the actively proliferating cells in the viable peripheral rim. The pharmacokinetics of paclitaxel in the ZD6126-treated tumor indicated greater accumulation in the peripheral rim of the tumor than the interior part.

The actual target in the tumor periphery might include endothelial cells, thus providing a rationale for combining a VDA with an anti-angiogenic agent (64). Rapid mobilization of circulating progenitor endothelial cells which home into the viable rim surrounding the necrotic area was reported in a tumor model of mice treated with the VDA OXi-4053, which was associated with the tumor vasculature (65).

## The Dual Face of Paclitaxel

Paclitaxel is one of the most widely used cytotoxic drugs, employed in the treatments of several neoplasms. This tubulin-binding agent promotes microtubule polymerization (at high concentrations) and impairs microtubule dynamics (at low concentrations), ultimately affecting mitosis, as well as other microtubule-dependent cell functions (66). The anticancer activity of paclitaxel extends beyond its cytotoxicity against tumor cells, since paclitaxel, and the tubulin-binding agents in general, also targets tumor stroma and vasculature inhibiting endothelial cell functions related to angiogenesis, at lower concentrations than those required for the cytotoxic activity (7, 49).

We have shown by *in vivo* optical, and dynamic contrast enhanced (DCE)-MRI imaging that paclitaxel can modify certain tumor vessel functions related to vascular perfusion and permeability (fractional plasma volume, fPV, and volume transfer coefficient, *k*_Trans_) (67). This was associated with increased tumor uptake of the antibody F8 (which selectively recognize perivascular EDA-fibronectin) conjugated to interleukin 2 (F8-IL2) (68). The use of antibody-based delivery of therapeutic agents in cancer therapy is beyond our scope and is covered by excellent reviews (59, 69).

Herein paclitaxel given before, but not after, F8-IL2 potentiated the latter’s antitumor activity on EDA-Fn expressing human melanoma xenografts. We attributed this to increased vascular permeability and perfusion at the tumor site, as the area perfused by Hoechst 33342 was larger in paclitaxel treated tumors than controls.

Although the mechanism of this effect of paclitaxel is far from clear, our findings are in line with other reports. Jain and coworkers proposed that taxane-induced tumor cell apoptosis reduced the IFP generated by neoplastic cell proliferation (solid stress) and decompressed tumor blood vessels. The increase in vessel diameter suggests that taxanes might improve tumor response by increasing the vascular surface area for the delivery of therapeutics (70). Paclitaxel and docetaxel lowered IFP and significantly increased albumin extravasation regardless of their cytotoxic activity, suggesting that these effects might be taxane-specific and related to the drugs’ pharmacodynamics (71).

The translational potential of these findings is substantiated by clinical studies. In breast cancer patients treated with neo-adjuvant chemotherapy, paclitaxel lowered IFP, and increased oxygenation (72); this suggests that at least these tumors would be best treated first with paclitaxel to reduce IFP and increase pO_2_ in order to improve the delivery of subsequent therapy, particularly of large molecules such as antibodies. However, the theory of solid stress in tumors only partially explains the biologic mechanisms by which taxanes boost the activity of combination therapy.

Two mechanisms have been proposed to explain the activity of co-administration of albumin-bound paclitaxel (Abraxane^®^, nab-paclitaxel) and gemcitabine in patients with pancreatic cancer (73). In a first study nab-paclitaxel increased the intratumoral concentration of gemcitabine in a mouse model of pancreatic ductal adenocarcinoma (PDA) (74). In a second study the nab-paclitaxel co-administered with gemcitabine caused tumor regression, due to a different mechanism, as gemcitabine was stabilized in the tumor by paclitaxel’s reduction in the levels of cytidine deaminase, the enzyme primarily responsible for gemcitabine metabolization, with no changes in overall drug delivery (75).

## Conclusion

The inhibition of tumor growth by drugs affecting the tumor vasculature has been achieved in preclinical and clinical studies. The combination of an angiogenesis inhibitor, namely bevacizumab, with chemotherapy, showed a benefit in patients with advanced disease, leading to increased interest in developing more effective ways to combine anti-angiogenic/vascular targeting agents with conventional chemotherapy. Morphological changes in the tumor microenvironment and vasculature are widely observed after angiogenesis inhibitors. However, the balance between lowering tumor microvessel density, impairing their function, and reducing/increasing drug uptake needs to be carefully considered in choosing the doses and schedule for combination settings.

Some preclinical studies have reported functional improvement in tumor blood perfusion after angiogenesis inhibitors, with increased tumor exposure to cytotoxic drugs. However, in other studies tumor vascular patency decreased, and hypoxia increased, with impaired cytotoxic drug uptake. The causal relationships between the effect on the microvasculature, the IFP reduction, and the improved trans-vascular transport are still not completely clear.

Nevertheless the combination of angiogenesis inhibitors with chemotherapy is almost always superior to single-drug treatment, indicating the beneficial effect of tumor cell starvation induced by angiogenesis inhibition. The order of administration of the two types of agents – anti-vascular and antitumor – is critical for a successful outcome and has far-reaching impact on the design of combination therapy. Careful optimization of drug scheduling and dosage is essential to maximize tumor response. Robust tumor pharmacodynamics and pharmacokinetics could help in fine-tuning drug timing and sequences so as ultimately to achieve a better outcome.

Monitoring the activity of angiogenesis inhibitors/vascular targeting agents is a significant practical challenge in the clinical setting, where non-invasive procedures such as imaging analysis and the detection of soluble biomarkers can be used to optimize the administration and determine the efficacy of combination regimens in patients.

## Conflict of Interest Statement

The authors declare that the research was conducted in the absence of any commercial or financial relationships that could be construed as a potential conflict of interest.
